# 2-Isopropyl-6-methyl­pyrimidin-4(3*H*)-one

**DOI:** 10.1107/S1600536810034276

**Published:** 2010-08-28

**Authors:** Madhukar Hemamalini, Hoong-Kun Fun

**Affiliations:** a X-ray Crystallography Unit, School of Physics, Universiti Sains Malaysia, 11800 USM, Penang, Malaysia

## Abstract

The mol­ecular structure of the title compound, C_8_H_12_N_2_O, indicates that 2-isopropyl-6-methyl­pyrimidin-4-ol (the enol–form) undergoes an enol-to-keto tautomerism during the crystallization process. The pyrimidin-4(3*H*)-one group is essentially planar, with a maximum deviation of 0.081 (1) Å for the O atom. In the crystal structure, symmetry-related mol­ecules are linked into centrosymmetic dimers *via* pairs of inter­molecular N—H⋯O hydrogen bonds, generating *R*_2_^2^(8) rings. These dimers are stacked along the *a* axis.

## Related literature

For applications of pyridinium derivatives, see: Condon *et al.* (1993 ▶); Maeno *et al.* (1990 ▶); Gilchrist (1997 ▶); Selby *et al.* (2002 ▶). For hydrogen-bond motifs, see: Bernstein *et al.* (1995 ▶). For the stability of the temperature controller used in the data collection, see: Cosier & Glazer (1986 ▶).
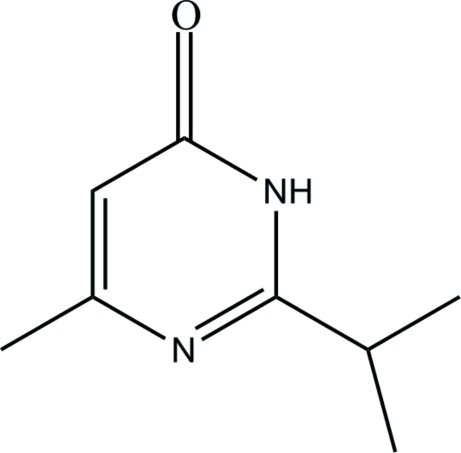


## Experimental

### 

#### Crystal data

C_8_H_12_N_2_O
*M* *_r_* = 152.20
Monoclinic, 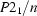

*a* = 4.8627 (2) Å
*b* = 22.6320 (8) Å
*c* = 7.4228 (3) Å
β = 96.495 (2)°
*V* = 811.66 (5) Å^3^
*Z* = 4
Mo *K*α radiation
μ = 0.08 mm^−1^
*T* = 100 K
0.74 × 0.14 × 0.07 mm

#### Data collection

Bruker SMART APEXII CCD area-detector diffractometer
Absorption correction: multi-scan (*SADABS*; Bruker, 2009 ▶) *T*_min_ = 0.940, *T*_max_ = 0.994
7806 measured reflections
2371 independent reflections
1958 reflections with *I* > 2σ(*I*)
*R*_int_ = 0.026

#### Refinement

*R*[*F* ^2^ > 2σ(*F* ^2^)] = 0.039
*wR*(*F* ^2^) = 0.103
*S* = 1.06
2371 reflections
148 parameters
All H-atom parameters refined
Δρ_max_ = 0.32 e Å^−3^
Δρ_min_ = −0.20 e Å^−3^

### 

Data collection: *APEX2* (Bruker, 2009 ▶); cell refinement: *SAINT* (Bruker, 2009 ▶); data reduction: *SAINT*; program(s) used to solve structure: *SHELXTL* (Sheldrick, 2008 ▶); program(s) used to refine structure: *SHELXTL*; molecular graphics: *SHELXTL*; software used to prepare material for publication: *SHELXTL* and *PLATON* (Spek, 2009 ▶).

## Supplementary Material

Crystal structure: contains datablocks global, I. DOI: 10.1107/S1600536810034276/lh5121sup1.cif

Structure factors: contains datablocks I. DOI: 10.1107/S1600536810034276/lh5121Isup2.hkl

Additional supplementary materials:  crystallographic information; 3D view; checkCIF report

## Figures and Tables

**Table 1:** Hydrogen-bond geometry (Å, °)

*D*—H⋯*A*	*D*—H	H⋯*A*	*D*⋯*A*	*D*—H⋯*A*
N2—H1*N*2⋯O1^i^	0.937 (15)	1.844 (14)	2.7809 (11)	178.7 (10)

Symmetry code: (i) 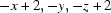.

**Fig. 1.**
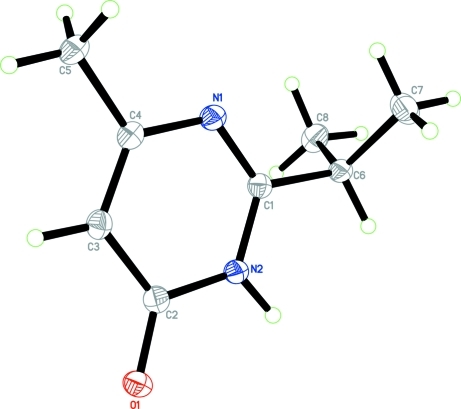
The molecular structure of the title compound. Displacement ellipsoids are drawn at the 50% probability level.

**Fig. 2.**
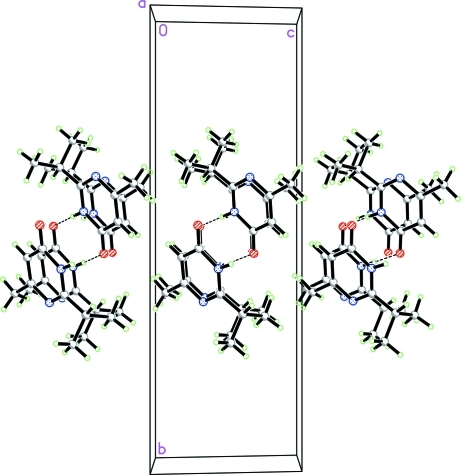
The crystal packing of the title compound, viewed approximately along the a-axis. Hydrogen bonds are shown as dashed lines.

**Fig. 3.**
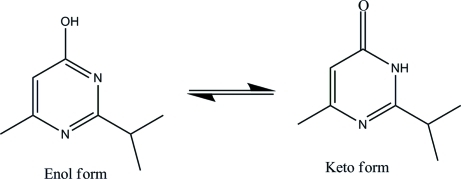
The title compound and the tautomeric form.

## supplementary crystallographic information

### Comment

Pyrimidine derivatives are very important molecules in biology and have many application in the areas of pesticide and pharmaceutical agents (Condon *et al.*, 1993). For example, imazosulfuron, ethirmol and mepanipyrim have been commercialized as agrochemicals (Maeno *et al.*, 1990). Pyrimidine derivatives have also been developed as antiviral agents, such as AZT, which is the most widely used anti-AIDS drug (Gilchrist, 1997). Recently, a new series of highly active herbicides of substituted azolylpyrimidines were reported (Selby *et al.*, 2002). Keeping in view of the importance of the pyrimidine derivatives, the title compound (I) was presented.

The title molecule, (Fig. 1), exists in the keto-form although 2-isopropyl-4-hydroxy-6-methylpyrimidine (the enol-form) was used for crystallization. This indicates the compound undergoes an enol-to-keto tautomerism during the crystallization process (Fig. 3). The C2═O1 bond length  is 1.2497 (11) Å. The pyrimidin-4(3*H*)-one group is essentially planar with a maximum deviation of 0.081 (1) Å for atom O1. In the crystal structure (Fig. 2), adjacent molecules are linked via pairs of intermolecular N—H···O hydrogen bonds to form dimers, generating *R*_2_^2^(8) rings (Bernstein *et al.*, 1995). These dimers are stacked along the *a*-axis.

### Experimental

Hot methanol solution (20 ml) of 2-isopropyl-4-hydroxy-6-methylpyrimidine (46 mg, Aldrich) was warmed over a heating magnetic stirrer for 5 minutes. The resulting solution was allowed to cool slowly at room temperature. Crystals of the title compound appeared from the mother liquor after a few days.

### Refinement

All H atoms were located in a difference Fourier map and refined freely.

### Crystal data

**Table:**

C_8_H_12_N_2_O	*F*(000) = 328
*M**_r_* = 152.20	*D*_x_ = 1.245 Mg m^−^^3^
Monoclinic, *P*2_1_/*n*	Mo *K*α radiation, λ = 0.71073 Å
Hall symbol: -P 2yn	Cell parameters from 2995 reflections
*a* = 4.8627 (2) Å	θ = 2.9–30.0°
*b* = 22.6320 (8) Å	µ = 0.08 mm^−^^1^
*c* = 7.4228 (3) Å	*T* = 100 K
β = 96.495 (2)°	Needle, colourless
*V* = 811.66 (5) Å^3^	0.74 × 0.14 × 0.07 mm
*Z* = 4	

### Data collection

**Table:**

Bruker SMART APEXII CCD area-detector diffractometer	2371 independent reflections
Radiation source: fine-focus sealed tube	1958 reflections with *I* > 2σ(*I*)
graphite	*R*_int_ = 0.026
φ and ω scans	θ_max_ = 30.1°, θ_min_ = 1.8°
Absorption correction: multi-scan (*SADABS*; Bruker, 2009)	*h* = −5→6
*T*_min_ = 0.940, *T*_max_ = 0.994	*k* = −26→31
7806 measured reflections	*l* = −10→10

### Refinement

**Table:**

Refinement on *F*^2^	Primary atom site location: structure-invariant direct methods
Least-squares matrix: full	Secondary atom site location: difference Fourier map
*R*[*F*^2^ > 2σ(*F*^2^)] = 0.039	Hydrogen site location: inferred from neighbouring sites
*wR*(*F*^2^) = 0.103	All H-atom parameters refined
*S* = 1.06	*w* = 1/[σ^2^(*F*_o_^2^) + (0.049*P*)^2^ + 0.163*P*] where *P* = (*F*_o_^2^ + 2*F*_c_^2^)/3
2371 reflections	(Δ/σ)_max_ < 0.001
148 parameters	Δρ_max_ = 0.32 e Å^−^^3^
0 restraints	Δρ_min_ = −0.20 e Å^−^^3^

### Special details

**Table:**

Experimental. The crystal was placed in the cold stream of an Oxford Cryosystems Cobra open-flow nitrogen cryostat (Cosier & Glazer, 1986) operating at 100.0 (1) K.
Geometry. All s.u.'s (except the s.u. in the dihedral angle between two l.s. planes) are estimated using the full covariance matrix. The cell s.u.'s are taken into account individually in the estimation of s.u.'s in distances, angles and torsion angles; correlations between s.u.'s in cell parameters are only used when they are defined by crystal symmetry. An approximate (isotropic) treatment of cell s.u.'s is used for estimating s.u.'s involving l.s. planes.
Refinement. Refinement of F^2^ against ALL reflections. The weighted R-factor wR and goodness of fit S are based on F^2^, conventional R-factors R are based on F, with F set to zero for negative F^2^. The threshold expression of F^2^ > 2σ(F^2^) is used only for calculating R-factors(gt) etc. and is not relevant to the choice of reflections for refinement. R-factors based on F^2^ are statistically about twice as large as those based on F, and R- factors based on ALL data will be even larger.

### Fractional atomic coordinates and isotropic or equivalent isotropic displacement parameters (Å^2^)

**Table:**

	*x*	*y*	*z*	*U*_iso_*/*U*_eq_	
O1	0.77782 (15)	0.03007 (3)	0.81600 (9)	0.02023 (18)	
N1	0.43265 (17)	−0.13343 (3)	0.84671 (10)	0.01613 (18)	
N2	0.75959 (17)	−0.06091 (3)	0.94759 (10)	0.01473 (17)	
C1	0.64511 (19)	−0.11546 (4)	0.95755 (12)	0.01455 (19)	
C2	0.6596 (2)	−0.01885 (4)	0.82081 (12)	0.0158 (2)	
C3	0.4232 (2)	−0.03768 (4)	0.70385 (12)	0.0169 (2)	
C4	0.32239 (19)	−0.09370 (4)	0.71759 (12)	0.0156 (2)	
C5	0.0868 (2)	−0.11656 (5)	0.58916 (13)	0.0190 (2)	
C6	0.7737 (2)	−0.15603 (4)	1.10544 (13)	0.01614 (19)	
C7	0.8468 (3)	−0.21559 (5)	1.02578 (15)	0.0249 (2)	
C8	0.5752 (2)	−0.16347 (5)	1.25028 (14)	0.0222 (2)	
H1N2	0.915 (3)	−0.0510 (7)	1.0283 (19)	0.034 (4)*	
H3A	0.338 (3)	−0.0101 (6)	0.6146 (18)	0.025 (3)*	
H5A	−0.064 (3)	−0.1299 (6)	0.655 (2)	0.036 (4)*	
H5B	0.011 (3)	−0.0864 (7)	0.503 (2)	0.044 (4)*	
H5C	0.151 (3)	−0.1505 (6)	0.5237 (19)	0.034 (4)*	
H6A	0.946 (3)	−0.1369 (5)	1.1613 (16)	0.018 (3)*	
H7A	0.922 (3)	−0.2424 (6)	1.124 (2)	0.031 (3)*	
H7B	0.984 (3)	−0.2117 (6)	0.9349 (19)	0.031 (4)*	
H7C	0.681 (3)	−0.2348 (6)	0.9648 (19)	0.036 (4)*	
H8A	0.661 (3)	−0.1874 (6)	1.3518 (19)	0.029 (3)*	
H8B	0.408 (3)	−0.1849 (6)	1.1978 (18)	0.031 (4)*	
H8C	0.519 (3)	−0.1249 (6)	1.2967 (19)	0.032 (4)*	

### Atomic displacement parameters (Å^2^)

**Table:**

	*U*^11^	*U*^22^	*U*^33^	*U*^12^	*U*^13^	*U*^23^
O1	0.0210 (4)	0.0159 (3)	0.0229 (4)	−0.0031 (3)	−0.0012 (3)	0.0032 (3)
N1	0.0159 (4)	0.0163 (4)	0.0157 (4)	−0.0010 (3)	0.0002 (3)	−0.0005 (3)
N2	0.0146 (4)	0.0143 (4)	0.0150 (4)	−0.0008 (3)	0.0005 (3)	0.0004 (3)
C1	0.0146 (4)	0.0146 (4)	0.0146 (4)	0.0003 (3)	0.0026 (3)	−0.0007 (3)
C2	0.0159 (4)	0.0161 (4)	0.0156 (4)	0.0005 (3)	0.0028 (3)	0.0007 (3)
C3	0.0165 (5)	0.0185 (4)	0.0153 (4)	0.0014 (3)	0.0006 (3)	0.0019 (3)
C4	0.0143 (4)	0.0185 (5)	0.0142 (4)	0.0005 (3)	0.0019 (3)	−0.0018 (3)
C5	0.0164 (5)	0.0227 (5)	0.0172 (4)	−0.0011 (4)	−0.0013 (3)	−0.0022 (4)
C6	0.0157 (4)	0.0151 (4)	0.0168 (4)	−0.0011 (3)	−0.0015 (3)	0.0009 (3)
C7	0.0301 (6)	0.0172 (5)	0.0260 (5)	0.0036 (4)	−0.0026 (4)	−0.0004 (4)
C8	0.0202 (5)	0.0268 (5)	0.0193 (5)	−0.0016 (4)	0.0012 (4)	0.0061 (4)

### Geometric parameters (Å, °)

**Table:**

O1—C2	1.2497 (11)	C5—H5B	0.978 (16)
N1—C1	1.3105 (12)	C5—H5C	0.978 (15)
N1—C4	1.3777 (12)	C6—C7	1.5297 (14)
N2—C1	1.3595 (12)	C6—C8	1.5332 (14)
N2—C2	1.3874 (12)	C6—H6A	0.990 (12)
N2—H1N2	0.937 (15)	C7—H7A	0.985 (14)
C1—C6	1.5114 (13)	C7—H7B	1.004 (14)
C2—C3	1.4254 (13)	C7—H7C	0.980 (15)
C3—C4	1.3672 (13)	C8—H8A	0.982 (14)
C3—H3A	0.968 (13)	C8—H8B	0.987 (14)
C4—C5	1.4972 (13)	C8—H8C	0.988 (15)
C5—H5A	0.973 (16)		
			
C1—N1—C4	116.83 (8)	H5A—C5—H5C	107.8 (12)
C1—N2—C2	123.08 (8)	H5B—C5—H5C	109.9 (12)
C1—N2—H1N2	119.2 (9)	C1—C6—C7	110.47 (8)
C2—N2—H1N2	117.7 (9)	C1—C6—C8	109.54 (8)
N1—C1—N2	123.11 (9)	C7—C6—C8	111.50 (8)
N1—C1—C6	119.97 (8)	C1—C6—H6A	107.5 (7)
N2—C1—C6	116.92 (8)	C7—C6—H6A	108.9 (7)
O1—C2—N2	120.02 (9)	C8—C6—H6A	108.9 (7)
O1—C2—C3	126.12 (9)	C6—C7—H7A	109.9 (8)
N2—C2—C3	113.86 (8)	C6—C7—H7B	112.5 (8)
C4—C3—C2	120.21 (9)	H7A—C7—H7B	109.4 (11)
C4—C3—H3A	121.2 (8)	C6—C7—H7C	110.8 (9)
C2—C3—H3A	118.6 (8)	H7A—C7—H7C	106.5 (12)
C3—C4—N1	122.85 (9)	H7B—C7—H7C	107.6 (11)
C3—C4—C5	121.86 (9)	C6—C8—H8A	110.7 (8)
N1—C4—C5	115.27 (8)	C6—C8—H8B	109.4 (8)
C4—C5—H5A	110.6 (9)	H8A—C8—H8B	106.8 (12)
C4—C5—H5B	112.3 (9)	C6—C8—H8C	111.6 (8)
H5A—C5—H5B	107.2 (12)	H8A—C8—H8C	109.3 (11)
C4—C5—H5C	108.9 (8)	H8B—C8—H8C	109.0 (11)
			
C4—N1—C1—N2	−1.11 (13)	C2—C3—C4—N1	2.79 (14)
C4—N1—C1—C6	178.69 (8)	C2—C3—C4—C5	−175.72 (8)
C2—N2—C1—N1	0.98 (14)	C1—N1—C4—C3	−0.79 (13)
C2—N2—C1—C6	−178.83 (8)	C1—N1—C4—C5	177.82 (8)
C1—N2—C2—O1	−178.41 (8)	N1—C1—C6—C7	52.88 (12)
C1—N2—C2—C3	0.98 (12)	N2—C1—C6—C7	−127.31 (9)
O1—C2—C3—C4	176.61 (9)	N1—C1—C6—C8	−70.32 (11)
N2—C2—C3—C4	−2.73 (13)	N2—C1—C6—C8	109.50 (9)

### Hydrogen-bond geometry (Å, °)

**Table:**

*D*—H···*A*	*D*—H	H···*A*	*D*···*A*	*D*—H···*A*
N2—H1N2···O1^i^	0.937 (15)	1.844 (14)	2.7809 (11)	178.7 (10)

Symmetry codes: (i) −*x*+2, −*y*, −*z*+2.
